# Robust Nonparametric Distribution Transfer with Exposure Correction for Image Neural Style Transfer

**DOI:** 10.3390/s20185232

**Published:** 2020-09-14

**Authors:** Shuai Liu, Caixia Hong, Jing He, Zhiqiang Tian

**Affiliations:** School of Software Engineering, Xi’an Jiaotong University, Xi’an 710049, China; hongcx@stu.xjtu.edu.cn (C.H.); smile_hj@stu.xjtu.edu.cn (J.H.); zhiqiangtian@mail.xjtu.edu.cn (Z.T.)

**Keywords:** robust nonparametric distribution transfer, exposure correction, neural style transfer

## Abstract

Image neural style transfer is a process of utilizing convolutional neural networks to render a content image based on a style image. The algorithm can compute a stylized image with original content from the given content image but a new style from the given style image. Style transfer has become a hot topic both in academic literature and industrial applications. The stylized results of current existing models are not ideal because of the color difference between two input images and the inconspicuous details of content image. To solve the problems, we propose two style transfer models based on robust nonparametric distribution transfer. The first model converts the color probability density function of the content image into that of the style image before style transfer. When the color dynamic range of the content image is smaller than that of style image, this model renders more reasonable spatial structure than the existing models. Then, an adaptive detail-enhanced exposure correction algorithm is proposed for underexposed images. Based this, the second model is proposed for the style transfer of underexposed content images. It can further improve the stylized results of underexposed images. Compared with popular methods, the proposed methods achieve the satisfactory qualitative and quantitative results.

## 1. Introduction

**Figure 1:**
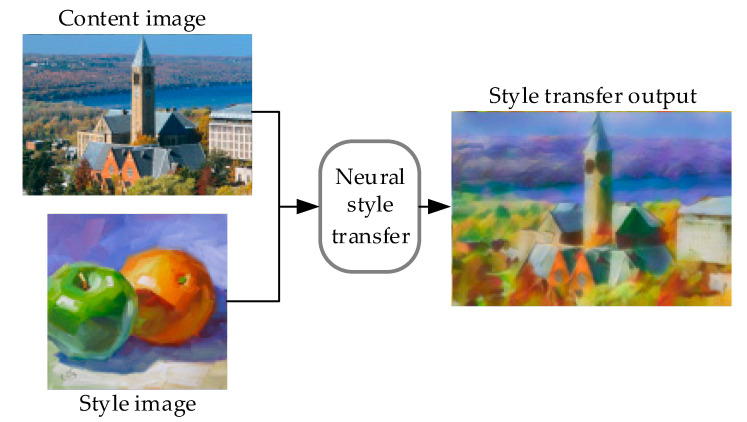
The processing of neural style transfer.

Style transfer seeks an artistic style reproduction on daily photos, enabling the photos retain the original content while presenting a prescribed artistic style [1,2,3]. The artistic style generally includes the color style and the texture style. By convention, the daily photo in style transfer is called content image, while the image who renders the artistic style is the style image. Style transfer makes artistic creation no longer distant for most people [4]. The process of neural style transfer is showed in Figure 1. The traditional artistic stylization algorithms are related to an area called non-photorealistic rendering (NPR), most of which transfer particular artistic styles [1,4]. In recent years, the development of deep learning [5,6,7] has made a breakthrough in style transfer [8,9,10,11]. The neural network enables the extraction of artistic styles, which makes it easier to convert daily photos into artistic photos [12]. For example, Gatys et al. [8] use the convolutional neural network (CNN) to reconstruct the content and style, and optimize the stylized image iteratively based on a loss function. From then on, CNN-based neural style transfer has become a hot topic [9,10,11,13,14].

For a long time, the research on neural style transfer mainly focused on two issues. It is hoped that the stylized images have a strong artistic sense and the model has good generalization for a wide application meanwhile. However, the application of style transfer is affected by many factors. Style images with large color differences from content images often generate errors during style transfer, while the inconspicuous details in low-illumination images also lead to unsatisfactory results.

This paper analyzes the influence of color dynamic range of input images on the stylized result and proposes two models to improve the spatial rationality of stylized image. Firstly, to solve the unreasonable space caused by the difference in the color dynamic ranges between content image and style image, we propose a neural style transfer model (RNDT) based on robust nonparametric distribution transfer. Then, a neural style transfer model combining exposure corrected and robust nonparametric distribution transfer (EC-RNDT) is proposed to further improve the stylized result of RNDT model for underexposed content images. Experimental results show that the style transfer methods based on robust nonparametric distribution transfer not only increased the spatial rationality, but also extended the application range.

The contributions of the proposed models are listed as follows: (1) We introduce a robust nonparametric distribution transfer algorithm into the style transfer model, which could improve the spatial rationality of the stylized image when the color styles of the content image and style image are quite different; (2) We introduce the adaptive exposure correction algorithm into the robust nonparametric distribution transfer to obtain the satisfactory stylized result for underexposed images.

## 2. Related Work

Since mid-1990s, different strategies have been used to preserve the structure of content images in the field of non-photorealistic rendering [1,4]. Song et al. [15] and Kolliopoulos [16] used a region-based algorithm to manipulate the geometry so as to control the local details. Hertzmann [17] proposed a stroke-based rendering to guide the virtual strokes placement on a digital canvas to endow the content image a prescribed style. Efros and Freemand [18] constrained the texture placement by the prior map exacted from content image, enabling the preservation of content and structure in content image. Even though the traditional artistic stylization can render a stylized image with original structure, it still lacks efficiency and flexibility.

The first neural style transfer (NST) model was proposed by Gatys et al. [8], which takes advantage of the deep convolutional neural network to extract high-level features and transfers them into arbitrary images. The loss function of this method consists of two parts, which are content loss and style loss. The content loss minimizes the distance of the content image and stylized image in feature space. The style loss is defined based on the difference between two Gram matrices from the style image and stylized image. Although the NST model addressed the limitations of the traditional artistic stylization methods, the details cannot be preserved due to the lack of low-level features.

Since the Gram-matrix based loss has limitations, many researchers found it is not the only choice for style representation. Li et al. [19] proposed a Maximum Mean Discrepancy (MMD) loss, which can be applied in NST. It is proved that optimizing Gram-based loss function equals to minimizing the MMD with a quadratic polynomial kernel. Li and Wand [20] introduced an MRF-based style loss function in patch level, which could preserve the details. However, it has limitation in depth information. Inspired by MRF-based style loss function [20], Liao et al. [21] proposed a deep image analogy method combining three parts, which are the idea of “image analogy” [22], the features exacted by CNN, and the idea of patch-wise matching. However, it works well only when the content image and style image have similar objects.

In addition, some research focuses on improving the stylized result through different information and strategies. Liu et al. [23] introduced a depth loss function based on the perceptual loss [24] to retain the overall spatial layout. Champandard [25] proposed a semantic-based algorithm by incorporating a semantic channel over the MRF loss [20], which leads to a more accurate semantic match. Li et al. [26] introduced an extra Laplacian loss, which introduces a constraint on low-level features. Gatys et al. [27] and Wang et al. [28] both utilized a coarse-to-fine procedure, enabling a large stroke size and high-resolution image stylization.

Based on the style transfer methods, many existing works focus on a specific application. Luan et al. [29] and Mechrez et al. [30] used a two-stage procedure to perform photorealistic style transfer. Chen et al. [31] proposed a bidirectional disparity loss for stereoscopic style transfer. Ruder et al. [32,33] applied style transfer to video by introducing a temporal consistency loss based on optical flow. Gatys et al. [27] proposed a color-preserving style transfer by performing a luminance-only transfer.

In recent years, the Generative Adversarial Networks (GAN) [34] have been widely used in image-to-image translation, which is similar to the neural style transfer. Isola et al. [35] utilized a conditional GAN [36] to implement the translation of label maps to street scenes, label maps to building facades, object edges to photos. Zhu et al. [37] used a cycle-consistent adversarial networks to translate the photograph and the paintings of famous artists. Image-to-image translation not only transfers the image styles, but also manipulates the attributes of the objects [35,37,38,39,40]. Therefore, the image-to-image translation can be seen as a generalization of the neural style transfer [41]. Besides, GANs learns the image styles from multiple images with similar style, while the neural style transfer only needs one content image and one style image.

In this paper, we analyze the factors that affect the rationality of the neural style transfer. Then two style transfer models are proposed based on robust nonparametric distribution transfer. Both style transfer models are verified in different application scenarios. Experimental results show that the proposed models can effectively improve the spatial rationality of the stylized images and preserve the structure information.

The rest of this paper will be organized as follows. We introduce our style transfer models in Section 3. In Section 4, the style transfer results of the proposed models and the existing models are analyzed. Finally, we conclude our work in Section 5.

## 3. Neural Style Transfer

In this section, we analyze two inevitable factors affecting the space rationality of the style transfer. One is the unreasonable space caused by the difference of the dynamic color ranges, and another one is underexposure. Both factors limit effectiveness of the neural style transfer models. Based on the analysis, we propose two neural style transfer models to ease the limitations and improve the effectiveness of the neural style transfer models. In Section 3.1, the RNDT model is presented to solve the problem of unreasonable space caused by the difference of the dynamic color ranges. In Section 3.2, the EC-RNDT style transfer model introduced the adaptive detail-enhanced multi-scale retinex into the RNDT model, which can simultaneously solve the problems of unreasonable space caused by the difference of the dynamic color ranges, and underexposure. Both methods can be utilized as the preprocessing of other neural style transfer models.

### 3.1. Robust Nonparametric Distribution Transferred Neural Style Transfer Model

Compared with the traditional artistic stylization, the neural style transfer method can work on any set of content images and style images. However, this approach does not yield a good stylized result for some input images. Since the colors in style images are usually more saturated than the content images, the color dynamic range of the style image is usually larger than that of the content image. We find that when the color dynamic range of the content image is much smaller than that of the style image, the artistic style texture is prone to being misplaced.

**Figure 2:**
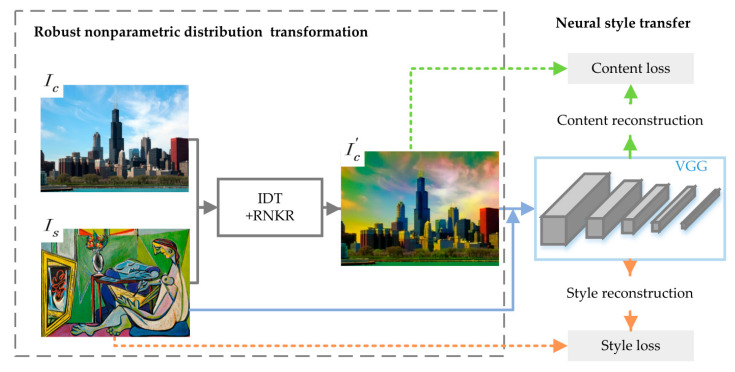
The structure of robust nonparametric distribution transferred neural style transfer.

To solve this problem, a neural style transfer model based on robust nonparametric distribution transfer is proposed. Before the style transfer, the color of the style image is transferred into the content image. This method is named robust nonparametric distribution transferred neural style transfer, which is shown in Figure 2.

For the robust nonparametric distribution transfer, it is constructed by introducing the robust nonparametric kernel regression (RNKR) [42] into the iterative Distribution Transform (IDT) model [43,44]. The IDT algorithm can change the color of all pixels into colors in the style image, while protecting the color continuity within the objects. When the color styles of the two input images are very different, and the color dynamic range of each image is very large, the IDT model can still get good results.

The IDT algorithm makes the color probability distribution of content image become that of the style image. Therefore, the color dynamic range of the content image is enhanced. Moreover, the boundaries of each object are more obvious. As a result, the content loss has a larger value at the boundary of the objects. Nonparametric kernel regression is a familiar tool to explore the underlying relation between response variable and covariates. The RNKR model [42] can control outlier effect through combining weighting and trimming to robustly achieve the nonparametric kernel regression. Based on this, RNKR is utilized to replace and obtain the optimal transport solution in IDT, when solving estimating equations in IDT.

The robust nonparametric distribution transfer procedure is presented by Equation (1),

(1) $$I_{c}^{\prime }\leftarrow t_{s}\left(I_{c},I_{s}\right)$$

where $I_{c}$ is the original content image. $I_{c}^{\prime }$ is the content image with the color of style image, which will be input into the feature extraction network. $t_{s}(\cdot )$ is a mapping function, which transforms the color distribution of $I_{c}$ into that of style image $I_{s}$. The input of $t_{s}(\cdot )$ is the content image $I_{c}$ and the style image $I_{s}$. A new image with the content of $I_{c}$ and the color of $I_{s}$ will be generated. The details of the $t_{s}(\cdot )$ are presented as Algorithm 1 [43,44].
**Algorithm 1** The details of the $t_{s}(\cdot )$**Initialization** of the source data $I_{c}$ and target $I_{s}$ For example in color transfer, $I_{c(j)}=(r_{\left(j\right)},g_{\left(j\right)},b_{\left(j\right)})$ where $r_{\left(j\right)},g_{\left(j\right)},b_{\left(j\right)}$ are the red, green, and blue components of pixel j. $k\leftarrow 0,I_{c}^{\left(0\right)}\leftarrow I_{c}$ **repeat**
(1)take one rotation matrix R(2)obtain the rotated samples: $I_{c(r)}\leftarrow RI_{c}^{\left(k\right)}$ and $I_{s(r)}\leftarrow RI_{s}$(3)project the samples on all axes j to get the marginal $f_{\left(j\right)}$ and $g_{\left(j\right)}$(4)for each axis j, find the 1D transformation $t_{s(j)}$ that matches the marginals $f_{\left(j\right)}$ and $g_{\left(j\right)}$ by using RNKR.(5)remap the samples $I_{c(r)}$ according to the 1D transformations by using RNKR. For example, a sample $\left(I_{c(1)},\ldots ,I_{c(N)}\right)$ is remapped into $\left(t_{1}(I_{c(1)}),\ldots ,t_{N}(I_{c(N)})\right)$, where N is the dimension of the samples.(6)rotate back the samples: $I_{c}^{\left(K+1\right)}\leftarrow R^{-1}I_{c(r)}$(7)k←k+1
**until** convergence on all marginals for every possible rotation The final one-to-one mapping $t_{s}(\cdot )$ is given by: $\forall j,I_{c(j)}^{}\to t_{s}\left(I_{c(j)}^{}\right)=I_{c(j)}^{\infty }$


In the neural style transfer model, VGG-19 [6] was used as a feature extractor to extract the high-level feature exaction for the input images and output images. The loss function of the proposed method consists of content loss and style loss. The content loss minimizes the content distance between the content image and the stylized image. The style loss is defined based on the difference between the high-level artistic style from the style image and the stylized image.

When the color dynamic range of the content image is much smaller than that of the style image, the artistic style textures tend to be placed incorrectly. The proposed RNDT model can improve the stylized effect in this case. In practical applications, the stylized result of the RNDT model is more spatially reasonable in this situation.

### 3.2. Exposure Corrected and Robust Nonparametric Distribution Transferred Neural Style Transfer Model

This section focuses on the daily photo stylization for cellphone users. Sometimes, photos are more likely to be underexposed. The underexposed photos usually have the following problems: (1) in the backlit area, the edge gradient of the object is smaller than normal area. The feature at the edge of the object is weakened so that the content loss function does not work well. (2) The color saturation in the backlit region is low, resulting in a small color dynamic range.

To solve these problems, an adaptive detail-enhanced multi-scale retinex (DEMSR) algorithm for exposure correction is proposed. Based on DEMSR, a neural style transfer model integrating exposure corrected and robust nonparametric distribution transfer is proposed.

#### 3.2.1. Adaptive Detail-Enhanced Multi-Scale Retinex Algorithm

Our proposed exposure correction algorithm for an underexposed content image consists of the following three steps.

Adaptive backlit region detection. The backlit region template detection is used to determine whether to modify the image. The adaptive detection method of backlit region is shown in Equation (2),

(2) $$M(x,y)=n_{0-1}\left(f\left(\lambda \times \left(L_{T}-L(x,y)\right)\right)\right)$$

where L is the lightness channel in CIELab space, $L_{T}$ is a threshold by which the image pixels are classed as backlit region or non-backlit region, λ is an empirical parameter that represents the degree of data polarization, f(u) is a function mapping the variate from range (−∞,+∞) to range (0,1), $n_{0-1}(z)=(z-\min(Z))/(\max(Z)-\min(Z))$ normalizes the pixels in the image. Here, f(u)=0.5/tanh(1+u), $L_{T}=70$, and λ=60.

According to [45], the guided filter can protect the edges of large objects in the image while blurring the image. Derived from a local linear model, the guided filter obtains the filtering output by learning the content of the guidance image, which can be the input image itself or another different image. Let F represent a guided filter whose window is 10% of the size of the content image. Then, the final backlit region template $M_{g}$ can be calculated by Equation (3).

(3) $$M_{g}=F*M$$

Light restoration module. Our exposure correction algorithm is developed based on the Multi-Scale Retinex (MSR) algorithm [46]. In the MSR algorithm, detail enhancement is performed on the luminance channel Y in YCbCr space. The algorithm estimates the natural light by blurring the luminance channel Y in three scales. Therefore, the reflected light R of the object itself can be estimated by Equation (4), while the exposure corrected luminance $Y^{MSR}$ is formulated by Equation (5).

(4) $$R_{i}=\log Y-\log L_{i},Y(x,y)\in [0,1]$$

(5) $$Y^{MSR}=n_{0-1}\left(\sum_{i=1}^{3}\frac{1}{3}R_{i}\right)$$

where $L_{i}=F_{i}\times Y,i=1,2,3$, $F_{i}$ are the Gaussian filters that estimate the natural light in three scales, the window sizes of $F_{1},\;F_{2},\;F_{3}$ are 0.8%, 6%, 15% of the content image, respectively. *Y^MSR^* is an exposure corrected image in the luminance channel which is calculated by the MSR algorithm.

In order to preserve the fine details after the robust nonparametric distribution transfer of the RNDT model, our method has made two improvements compared to the MSR algorithm. (1) Removing the “Halo” phenomenon to protect the reality of the boundary of the backlit area. For this purpose, the Gaussian filter used in Equation (4) is changed into a guided filter. The guided filter can protect the edge of the backlit region, while estimating the natural light *L_i_*; (2) Enhancing the edge and stereoscopy of the object inside the backlit area. The idea of the Laplacian filter [47] is used to enhance the edge of the object in the enhanced backlit area by Equation (6),

(6) $$Y^{DEMSR}=Y^{MSR}+\left(Y-F_{4}*Y\right)$$

where *F*_4_ is a Laplacian filter whose window is 0.4% of the content image. *Y^DEMSR^* is the exposure corrected image in the luminance channel obtained by the DEMSR algorithm. After enhancing the details, a gain compensation on *Y^DEMSR^* will be conducted for the balance of the three channels. Finally, the YCbCr space is converted to the RGB space to obtain the backlit enhanced image $I^{DEMSR}$.

Fusion to obtain exposure correction image. Once the backlit enhanced image is obtained, the non-backlit region is filtered by the backlit region template. The enhanced backlit area is fused with the normal area to obtain the final exposure correction image $I_{ec}$ by Equation (7).

(7) $$I_{ec}=M_{g}\cdot I^{DEMSR}+\left(1-M_{g}\right)\cdot I_{c}$$

The final exposure correction image will be input to the RNDT model for getting the stylized image.

#### 3.2.2. EC-RNDT Model

**Figure 3:**
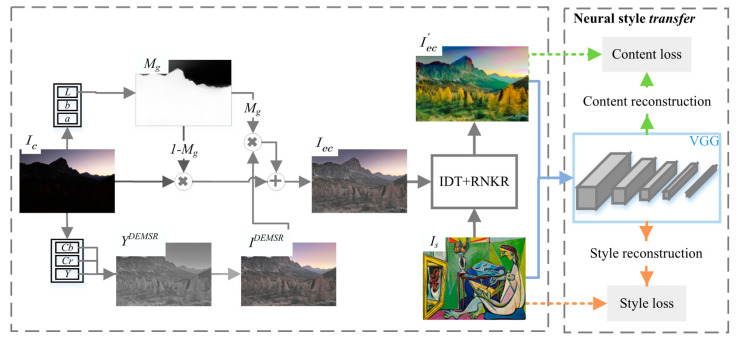
The structure of neural style transfer model using EC-RNDT.

Figure 3 shows the proposed EC-RNDT neural style transfer model combining adaptive DEMSR algorithm and RNDT algorithm. The exposure correction and color distribution transform include three steps. The first step is an automatic detection of whether the image is underexposed. Second, the light restoration and the enhancement of edge details are performed. Finally, the exposure correction image $I_{ec}$ is input into the RNDT model.

(8) $$I_{ec}^{\prime }\leftarrow t_{s}\left(I_{ec},I_{s}\right),$$

where $I_{ec}^{\prime }$ is the exposure correction image with the color of style image, which will be the content image of neural style transfer.

## 4. Experimental Results

### 4.1. Experiment Details

The RNDT algorithm and DEMSR algorithm are implemented by Matlab, and the style transfer models use the Lua language and Torch framework. The algorithm was implemented in Python with developed codes subject to the PyTorch framework. The algorithm ran on an Ubuntu system with an Intel Xeon E5-2620 CPU (2.1 GHz) and with a 64 GB memory. Our code used a GTX 1080 Ti GPU with 11 GB memory. The implementation was not optimized and did not use multithread and parallel programming. Computing by using the test images in this section, the average computational cost of RNDT is 1.1 s, and the average computational cost of EC-RNDT is 4 s. In the experiments, all the style transfer models run in the same environment, and all the hyper-parameters are unchanged in the comparison experiments.

It is a subjective task to evaluate the quality of the style transfer [3,4]. Therefore, most of evaluations of neural style transfer models are qualitative [48,49]. The most common method is to qualitatively compare results of style transfer methods by putting stylized images side by side [11,12,13,14,21,24,29]. Besides showing stylized images, user study is also used for evaluation [9,14,29]. The typical setup is to recruit some users, show them stylized images of different models, and ask them which results they prefer. The qualitative results will be presented in Section 4.2, Section 4.3, Section 4.4 and Section 4.5, while the quantitative results of user study are shown in Section 4.6.

### 4.2. Style Transfer Results of RNDT Model

**Figure 4:**
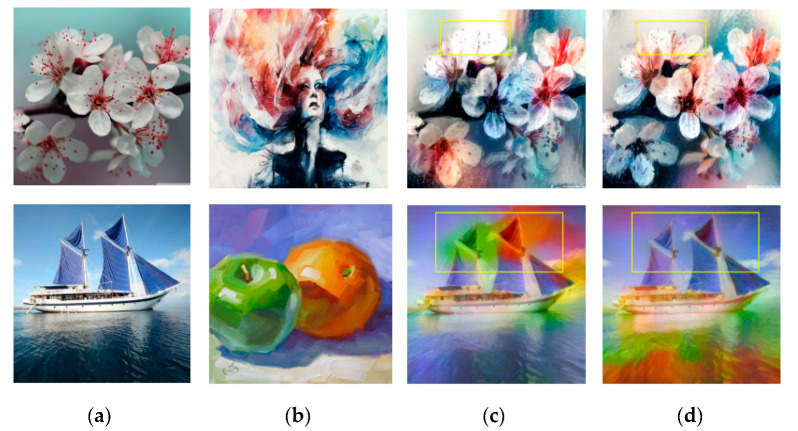
The style transfer results of Gatys et al. and RNDT model: (**a**) content image; (**b**) style image; (**c**) Gatys et al.; (**d**) RNDT.

For the comparison experiments, the models of Gatys et al. [8], Huang et al. [11], Li et al. [9], Liao et al. [21], and Johnson et al. [24] were used as the baselines. The results of these five baseline models and our RNDT model were compared to verify the superiority of the proposed method. The results of Gatys et al. [8] and RNDT model are shown in Figure 4. In the first result of Gatys et al., the color of flowers is similar with the background (yellow box), resulting in an ambiguous edge of the flower. It also has ambiguous edge for the sail in the second row (yellow box). However, after the robust nonparametric distribution transfer of content image, the proposed model can generate more reasonable stylized results. The distribution of color is more reasonable, and the boundaries of the objects are much clearer than the left result.

**Figure 5:**
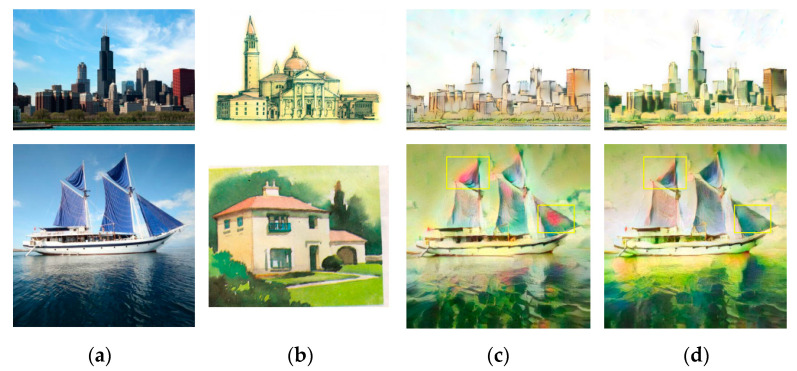
The style transfer results of Huang et al. and RNDT model: (**a**) content image; (**b**) style image; (**c**) Huang et al.; (**d**) RNDT.

**Figure 6:**
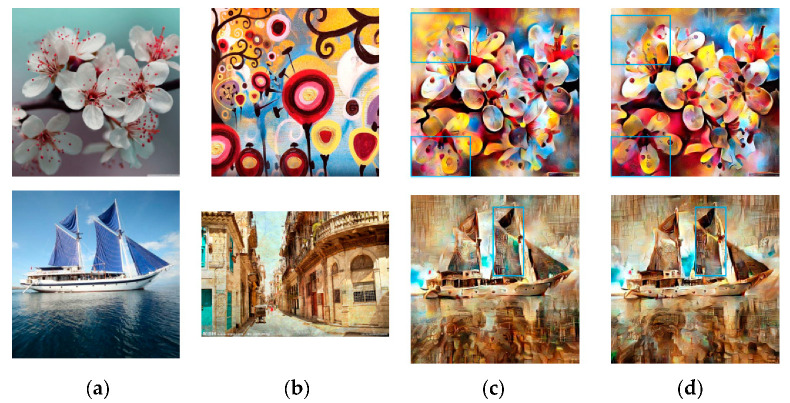
The style transfer results of Li et al. and RNDT model: (**a**) content image; (**b**) style image; (**c**) Li et al.; (**d**) RNDT.

**Figure 7:**
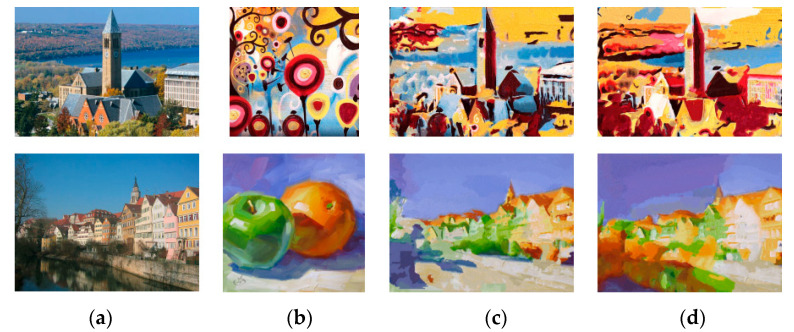
The style transfer results of Liao et al. and RNDT model: (**a**) content image; (**b**) style image; (**c**) Liao et al.; (**d**) RNDT.

**Figure 8:**
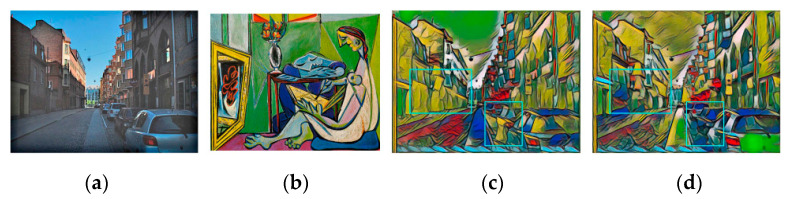
The style transfer results of Johnson et al. and RNDT model: (**a**) content image; (**b**) style image; (**c**) Johnson et al.; (**d**) RNDT.

Figure 5 shows the stylized results of Huang et al. [11] and RNDT model. In the first row, the model of Huang et al. does not transfer the primary color of the buildings in the style image into the content image. At the same time, the model does not transfer the red color in a proper location in the second row, and the spatial distribution of colors in the result is unreasonable. The proposed robust nonparametric distribution transfer solves these problems. In the first row, the RNDT model transfers the yellow color in the style image to the output, and preserves the structures. In the second result of RNDT model, the colors inside the sails are more continuous. The stylized results of our method have clearer edges than model of Huang. Similarly, other three comparison results are presented in Figure 6, Figure 7 and Figure 8.

**Figure 9:**
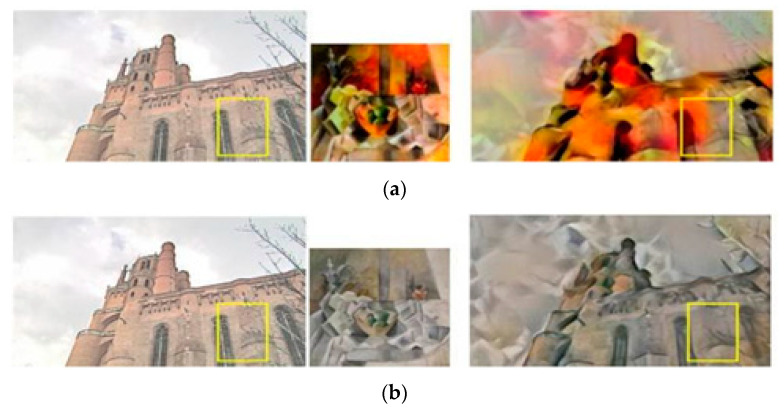
The impact of different color dynamic ranges between the content image and style image on the results: (**a**) when the color dynamic range of $I_{c}$ is smaller than $I_{s}$, the texture is misplaced; (**b**) when the color dynamic range of $I_{c}$ is close to $I_{s}$, the texture is placed correctly.

When the color dynamic range of the content image is much smaller than that of the style image, the artistic style texture is prone to be misplaced. An experiment was performed to show that the existing neural style transfer models suffer from this problem. The results are shown in Figure 9. The first row shows the results where the color dynamic range of the content image is smaller than that of the style image. We can see that the texture of stylized image is misplaced (yellow box). Meanwhile, when the color dynamic ranges of the content image and style image are close, as shown in the second row of the figure, the texture is placed correctly.

**Figure 10:**
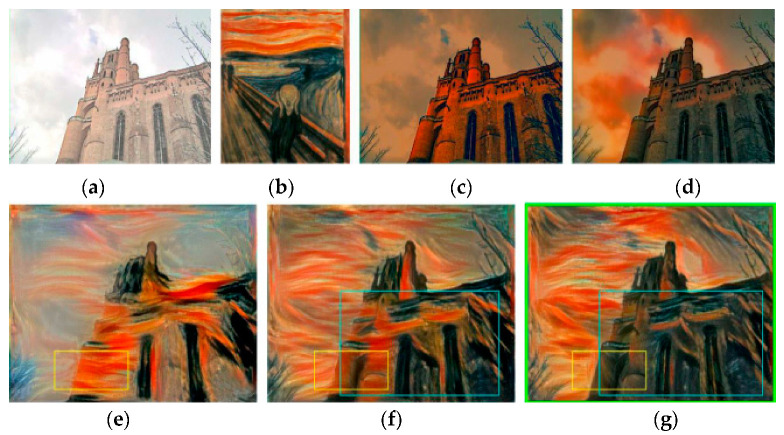
Comparison results: (**a**) content image; (**b**) style image; (**c**) $I_{c}^{\prime }$ based on Reihard; (**d**) $I_{c}^{\prime }$ based on RNDT; (**e**) transfer result of Gatys; (**f**) result based on Reihard; (**g**) result based onRNDT.

To evaluate the impact of the RNDT algorithm, an experiment was performed. The experiment results are shown in Figure 10, where $I_{c}^{\prime }$ is the content image with the color of style image. When the color dynamic range of content image is far smaller than that of style image, as shown in Figure 10a,b, the method of Gatys et al. [8] cannot maintain the spatial structure of the content image. As shown in the yellow box of Figure 10e, sky area and building area are wrongly mixed together, while the RNDT method could overcome this defect. As shown in the yellow box of Figure 10f,g, the sky and building are distinguished, which benefit from the color pre-transformation. Furthermore, we find that the result based on RNDT is better than that of using Reinhard [50]. As shown in the blue box of Figure 10f,g, the result based on RNDT has more uniform color inside the building area. It could be explained based on the intermediate results of color pre-transformation, which are shown in Figure 10c,d. The linear color transformation algorithm of Reinhard tends to produce an excessive discoloration. However, the nonlinear RNDT algorithm has no excessive color change, which could maintain the spatial structure of the object.

### 4.3. Results of DEMSR Algorithm

**Figure 11:**
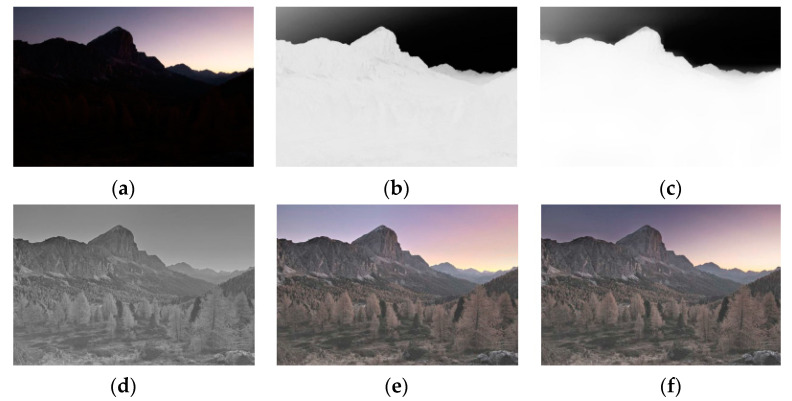
The illustration of the Adaptive Detail-Enhanced Multi-Scale Retinex algorithm: (**a**) content image; (**b**) initial backlit region template; (**c**) final backlit region template; (**d**) Y channel of backlit enhanced image; (**e**) backlit enhanced image; (**f**) exposure correction image.

To illustrate the effectiveness of the DEMSR algorithm, the intermediate results are shown in Figure 11. These results correspond to different exposure correction steps in DEMSR, respectively. More specifically, Figure 6a shows an underexposed content image. Figure 11b,c shows the outputs of the adaptive backlit region detection. Figure 11d,e shows the results of the light restoration module. Finally, Figure 11 is the exposure correction image of the proposed DEMSR algorithm. Through the DEMSR algorithm, the light was restored and the details in the backlit region were enhanced.

**Figure 12:**
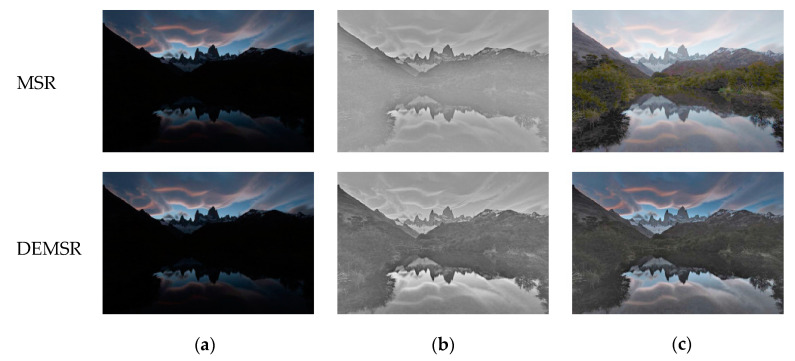
The comparison between MSR and the proposed DEMSR algorithms: (**a**) source image; (**b**) Y channel of backlit enhanced image; (**c**) exposure correction image.

The light restoration results of the MSR algorithm and our DEMSR algorithm are compared in Figure 12. Since the object edge inside the backlit region was enhanced in the DEMSR, the output of DEMSR can show the details more clearly than the MSR algorithm.

To further evaluate the performance of MSR and the proposed DEMSR algorithms, two quantitative metrics are adopted, which are peak signal-to-noise ratio (PSNR) and structural similarity (SSIM) [51]. The bigger values of these two metrics mean better performance. The PSNR obtained from MSR algorithm is 8.35, while the PSNR obtained from DEMSR is 11.85. The MSR algorithm gets a SSIM of 0.26, while the proposed DEMSR gets a SSIM of 0.39. Therefore, the proposed DEMSR algorithm performs better than the MSR algorithm.

**Figure 13:**
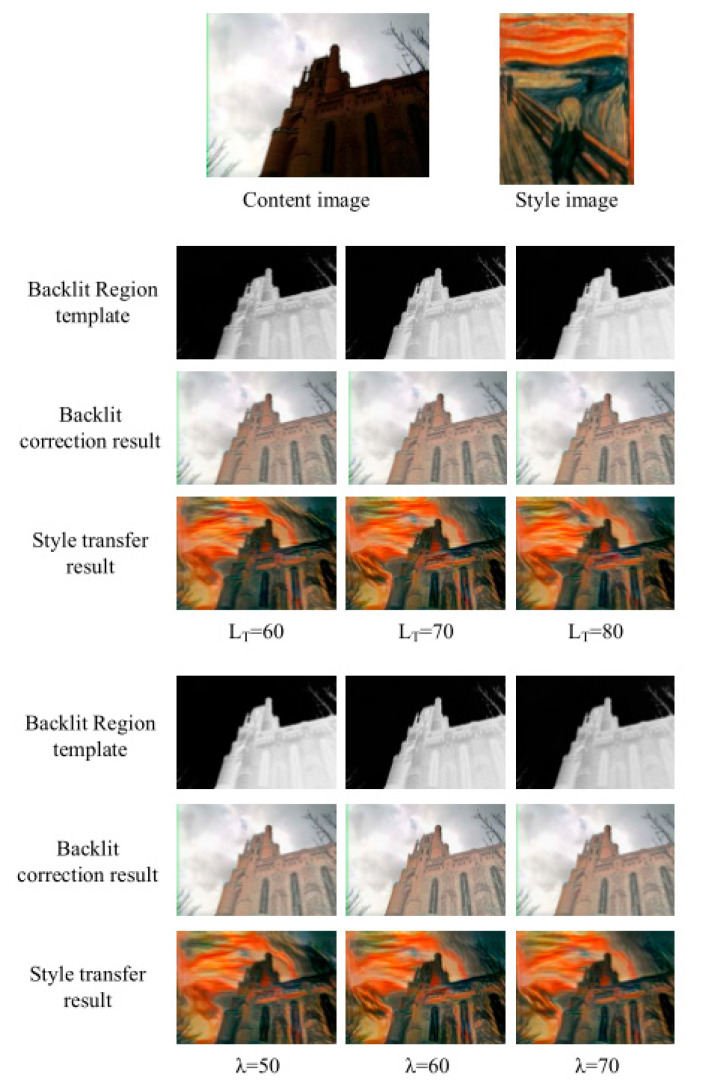
The influences of the backlit-region-detection parameters *L_T_* and *λ*.

The adjustment of parameters $L_{T}$ and λ in the backlit region detection determines the effect of exposure correction. Therefore, we performed an experiment to evaluate the influences of these two parameters. The results obtained based on different values of the parameters are shown in Figure 13. From the figure, we can see that there is only a slight change between different style transfer results. Therefore, the proposed method is robust to the parameters $L_{T}$ and λ in a fixed range, e.g., $L_{T}$ varies from 60 to 80, while λ varies from 50 to 70.

### 4.4. Style Transfer Result of EC-RNDT Model

#### 4.4.1. Comparison of RNDT Model and EC-RNDT Model

In order to show the effect of the DEMSR algorithm, we compared the results of the RNDT model with the EC-RNDT model.

**Figure 14:**
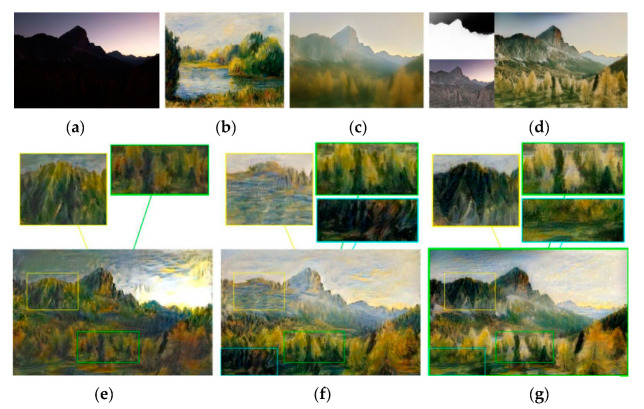
The results of Gatys et al., RNDT and EC-RNDT model on the underexposed content image: (**a**) content image; (**b**) style image; (**c**) $I_{c}^{\prime }$; (**d**) *M_g_,* $I_{ec}^{}$, $I_{ec}^{\prime }$; (**e**) Gatys et al.; (**f**) RNDT; (**g**) EC-RNDT.

In Figure 14, the output image of Gatys et al. [8], RNDT, and EC-RNDT model on the underexposed content image are shown, respectively. Figure 8a is the underexposed content image and Figure 8b is the style image. Figure 8c is the result of robust nonparametric distribution transfer in the RNDT model. In Figure 8d, $M_{g}$ is the backlit template, $I_{ec}$ is the result of DEMSR algorithm, and $I_{ec}^{\prime }$ is the result of the robust nonparametric distribution transfer in the EC-RNDT model. Figure 8e–g shows the stylized results of Gatys et al., RNDT model, and EC-RNDT model, respectively.

In Figure 14, the tree and hill in the stylized result of Gatys et al. have the same color and texture (yellow and green boxes), which makes the structures unclear. In the stylized result of RNDT, the texture of the tree and hill become different due to the robust nonparametric distribution transfer. However, the details in the stylized image are still unclear, such as the boundary between grasses and trees (the blue box), the details inside the hill (the yellow box), and the boundary between different trees (the green box). The EC-RNDT model further improves the stylized result by using the exposure correction. Since the DEMSR algorithm enhances the details in the backlit region, the EC-RNDT model can preserve the fine details in the content image. Benefiting from the DEMSR algorithm, the EC-RNDT model can better protect the spatial rationality of the stylized image than RNDT model when the content image is underexposed.

#### 4.4.2. Comparison between Baselines and EC-RNDT Mode

In order to show the superiority of the proposed EC-RNDT model, we compared four baseline models [8,11,21,24] with the EC-RNDT model.

In the experiments, the models of Gatys et al. [8] and Huang et al. [11] were used as the baselines. The stylized results of baselines [8,11] and EC-RNDT model on an underexposed content image were compared. These results show the effectiveness of the exposure correction and robust nonparametric distribution transfer.

**Figure 15:**
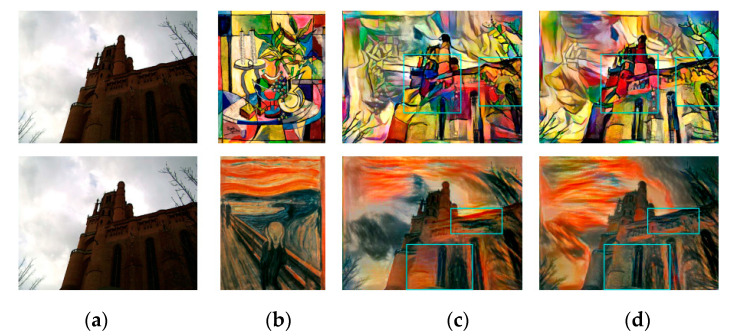
The style transfer results of Gatys et al. and EC-RNDT model: (**a**) content image; (**b**) style image; (**c**) Gatys et al.; (**d**) EC-RNDT.

The stylized results of Gatys et al. [8] and EC-RNDT model are shown in Figure 15. As shown in the green box, the stylized results of Gatys et al. loose some details in the building, resulting in an ambiguous structure of the building. Comparing Figure 15c,d, it is easily found that the EC-RNDT model can render the color and preserve the structure better. It can be attributed to exposure correction preforming before the style transfer, which can learn and preserve the fine details in the backlit region well.

**Figure 16:**
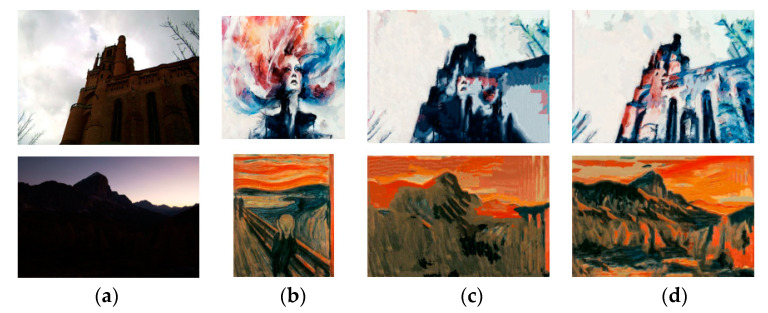
The style transfer results of Liao et al. and EC-RNDT model: (**a**) content image; (**b**) style image; (**c**) Liao et al.; (**d**) EC-RNDT.

**Figure 17:**
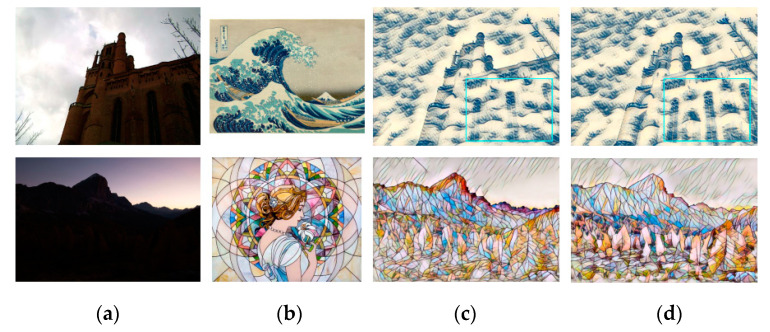
The style transfer results of Johnson et al. and EC-RNDT model: (**a**) content image; (**b**) style image; (**c**) Johnson et al.; (**d**) EC-RNDT.

**Figure 18:**
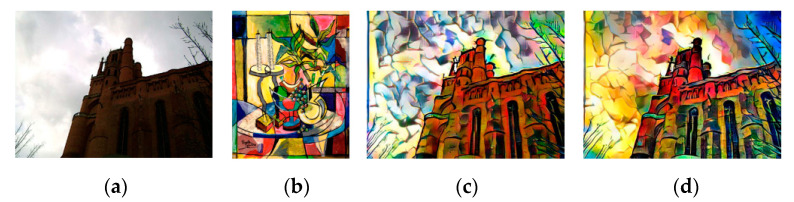
The style transfer results of Huang et al. and EC-RNDT model: (**a**) content image; (**b**) style image; (**c**) Huang et al.; (**d**) EC-RNDT.

The stylized results of Liao et al. and EC-RNDT model are shown in Figure 16. From the first row, it is obvious that the structures of the building obtained by EC-RNDT are much clearer than that of Huang et al. Meanwhile, the EC-RNDT model renders more colorful results while Huang et al. cannot effectively transfer all the colors of the style image. In the second row, the trees and the reflection in the stylized image of Huang et al. have similar color and texture, which makes the image content blurry. For EC-RNDT model, the different kinds of object have different colors and textures. Therefore, the EC-RNDT model can effectively retain the image content, and transfer the texture and color styles. Similar comparison results are presented in Figure 17 and Figure 18.

### 4.5. Color-Preserved Style Transfer

**Figure 19:**
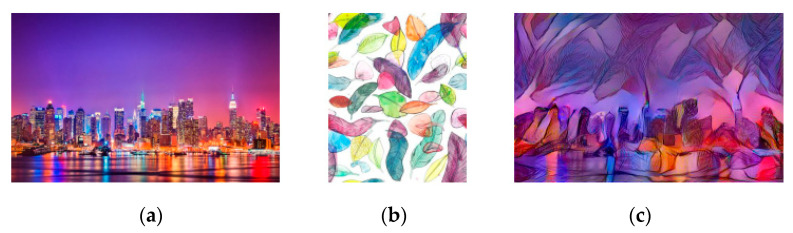
The result of color-preserved RNDT model: (**a**) content image; (**b**) style image; (**c**) color-preserved result.

Aforementioned sections showed the color transfer of the content image. Under certain circumstances, style transfer is hoped to preserve the color of the content image but transfer the texture style. In this case, the RNDT model can achieve this demand through a slight modification. The input image of RNDT model performs a role exchange, which can realize the color-preserved transfer. That is to say, before the style transfer model, the color of style image is changed into the color of the content image. Therefore, the style transfer will only transfer the texture style of the style image. The results of color-preserved RNDT model are shown in Figure 19.

### 4.6. Quantitative Results

A user study was performed to further valid the superiority of the proposed models. In the user study, we designed a webpage including 60 groups of outputs, which were viewed by participants. Each group contains two stylized image of baseline models and one stylized image from the proposed model. Participants were asked to choose the one he or she likes better. The results of user study are shown in Table 1 and Table 2. The values in the table presents the preference (the percentage of the votes) on the stylized results of different models. It shows that the stylized results of proposed models are better than the stylized results from baseline models. From the user study, we conclude that the proposed models can render more artistic and popular results.

As a form of preprocessing, the proposed model can improve the performances of the corresponding style transfer method without depending on any specific neural style transfer model. To evaluate this, two quantitative metrics were adopted, which are deception rate [52] and FID core [53]. As defined in [52], deception rate is proposed for an automatic evaluation of style transfer results. It is calculated as the fraction of generated images which were classified by the network as the artworks of an artist for which the stylization was produced. FID score evaluates the style transfer results by measuring the distance between the generated distribution and the real distribution [53]. The higher deception rate means the better performance, while the lower FID score represents the better performance. Gatys [8] and Huang [11] were used as the compared methods. Then the proposed model was used as preprocessing of Gatys [8] and Huang [11]. The deception rate and FID core of different methods are shown in Table 3, Table 4, Table 5 and Table 6 separately. From the tables, we can see that the proposed method outperforms two state-of-the-art methods.

## 5. Conclusions

In this paper, we propose two models for image neural style transfer. The first model named as RNDT transfers the color of the content image to that of the style image, which makes the spatial structure of the stylized image more reasonable. The second model introduces the DEMSR exposure correction algorithm to enhance the details in the underexposed images, which can preserve the fine details in the stylized images. Experimental results show that the models have improved the spatial rationality and the artistic sense of the stylized images. The proposed models could be applied in several situations, which include underexposed images, large color-range images, and color-preserved style transfer. Therefore, our models have a high application value.

The proposed RNDT and EC-RNDT models do not depend on any specific neural style transfer model. They work for the style transfer methods that only need one style image. They can improve the performances of the corresponding style transfer methods as a method of preprocessing.

The proposed models achieve encouraging performance in generating stylized images. However, we have to note that our approach still has limitation. The proposed models are not end-to-end, which is not efficient. Therefore, developing an efficient end-to-end style transfer method is our future work.

## Figures and Tables

**Table 1:** The preference (%) of stylized images of baseline models and RNDT model when the color dynamic ranges of two input images are much different.

Model	Gatys et al.	Huang et al.	RNDT
Preference	33.60	24.11	42.49

**Table 2:** The preference (%) of stylized images of baseline models and EC-RNDT model on the underexposed content images.

Model	Gatys et al.	Huang et al.	EC-RNDT
Preference	31.21	20.95	47.84

**Table 3:** Deception rate on style transfer in terms of different methods. Higher values indicate better performance.

Model	Gatys [8]	Ours + Gatys
Deception rate	0.43	0.49

**Table 4:** Deception rate on style transfer in terms of different methods. Higher values indicate better performance.

Model	Huang [11]	Ours + Huang
Deception rate	0.31	0.32

**Table 5:** FID score on style transfer in terms of different methods. Lower score indicates better performance.

Model	Gatys [8]	Ours + Gatys
FID score	265.3	262.8

**Table 6:** FID score on style transfer in terms of different methods. Lower score indicates better performance.

Model	Huang [11]	Ours + Huang
FID score	245.8	241.7
